# Supplementary material for: FGF4 initiates X-chromosome inactivation via activating YY1 and prompting loss of pluripotency factors
Source: EMBO J. 2026 Mar 25;45(9):3056–72. doi: 10.1038/s44318-026-00722-2 (PMC13144533; doi:10.1038/s44318-026-00722-2)
Supplement: Supplementary file 2 — Appendix [file 44318_2026_722_MOESM2_ESM.pdf]

Supporting Information

FGF4 initiates X-chromosome inactivation via activating YY1 and prompting loss of pluripotency factors

Lizhu Ma[#], Wei Fu[#, &], Lei An[#], Qianying Yang, Ruiqi Hai, Guangyin Xi, Yinjuan Wang, Juan Liu, Chao Zhang, Fu Yao, Zhenni Zhang, Xiaodong Wang, Jianhui Tian[*]

State Key Laboratory of Animal Biotech Breeding; National Engineering Laboratory for Animal Breeding; Key Laboratory of Animal Genetics, Breeding and Reproduction of the Ministry of Agriculture and Rural Affairs; College of Animal Science and Technology, China Agricultural University, No.2 Yuanmingyuan West Road, Beijing, China.

*Corresponding author: Jianhui Tian

Email: tianjh@cau.edu.cn

[#] These authors contributed equally.

[&] Present address: Key Laboratory of Qinghai-Tibetan Plateau Animal Genetic Resource Reservation and Utilization, Southwest Minzu University, Ministry of Education, Chengdu, China

Table of Contents

Appendix Figure S1 2
Appendix Figure S2 3
Appendix Figure S3 5
Appendix Figure S4 6
Appendix Figure S5 7
Appendix Figure S6 9
Appendix Figure S7 10
Appendix Table S1.. 12

Appendix Figure S1

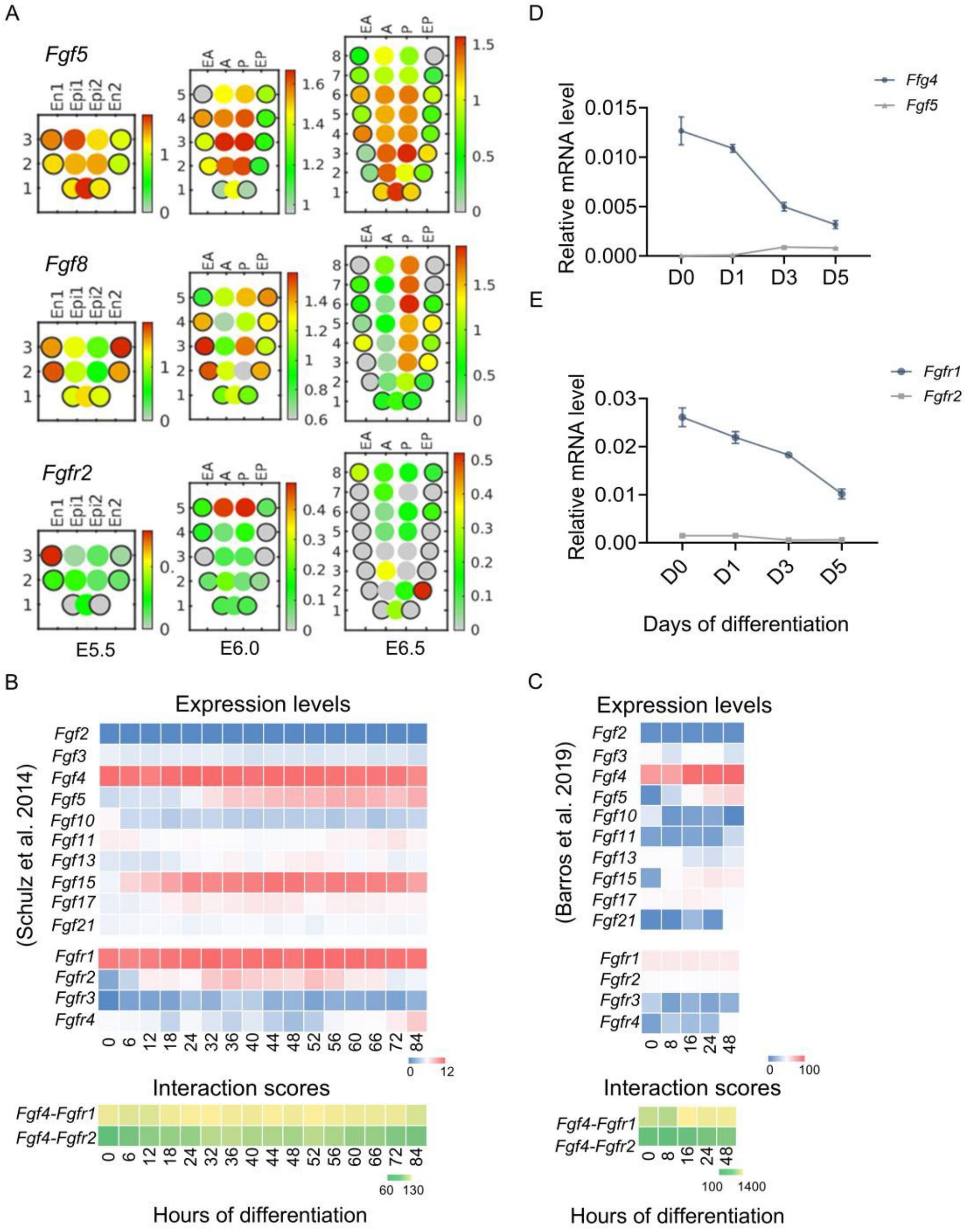

Appendix Figure S1. (A) The presentation of the spatial pattern of gene expression of *Fgf5*, *Fgf8* and *Fgfr2* in gastrulating mouse embryos. Data source: eGastrulation (http://egastrulation.sibcb.ac.cn). (B and C) Dynamic expression levels of *Fgfs* (B and C, upper panel), *Fgfrs* (B and C, upper panel) and Interaction score (B and C, down panel) at different time points of differentiation using publicly available transcriptome data(Barros et al, 2019; Schulz et al, 2014). (D and E) The relative mRNA expression of *Fgf4* (D), *Fgf5* (D), *Fgfr1* (E) and *Fgfr2* (E) genes in WT ES cells on day 0, 1, 3 and 5 of differentiation. Data was represented as means ± SEM; *n* = 3.

Appendix Figure S2

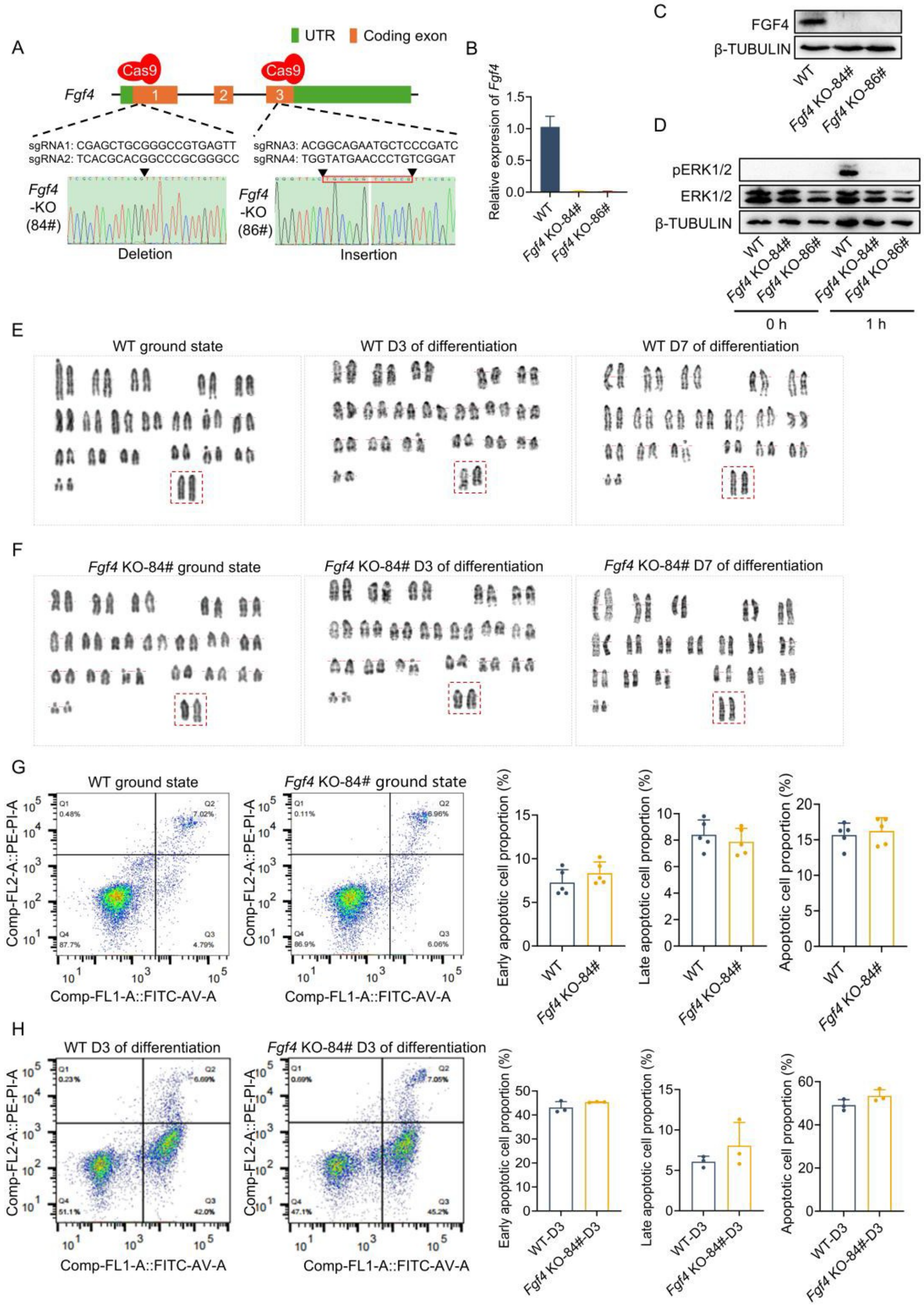

Appendix Figure S2. (A) Flow diagram of CRISPR/CAS9n-based knockout of *Fgf4*. (B and C) levels of *Fgf4*/FGF4 in *Fgf4* KO-84# or *Fgf4* KO-86# ES cells detected by RT-qPCR (B) and Western blots (C) respectively. (D) Western blot analysis of ERK and pERK in *Fgf4* KO-84# or *Fgf4* KO-86# ES cells at 0 and 1 h of differentiation, showing a functional knockout of *Fgf4*. Before the differentiation process, cells suffered MEK/ERKi (PD0325901, 1 μM) treatment in SCM medium for 24 h to completely inhibit the MEK/ERK activities. Then, the medium was

replaced by differentiation medium N2B27 without LIF or FBS for 1 h treatment. (E and F) The karyotype analysis of WT, Fgf4 KO-84# ES cells at the ground state, or day 3 and 7 of differentiation. The boxed region highlighted by the red dotted line indicates X chromosomes. (G and H) The fluorescence-activated cell sorting (FACS) pictures (F and G, left panel) and quantification results (F and G, right panel) of WT and *Fgf4* KO-84# cells on ground state and day 3 of differentiation; (B, G, H) Data was represented as means ± SEM; *n* = 3-5.

Appendix Figure S3

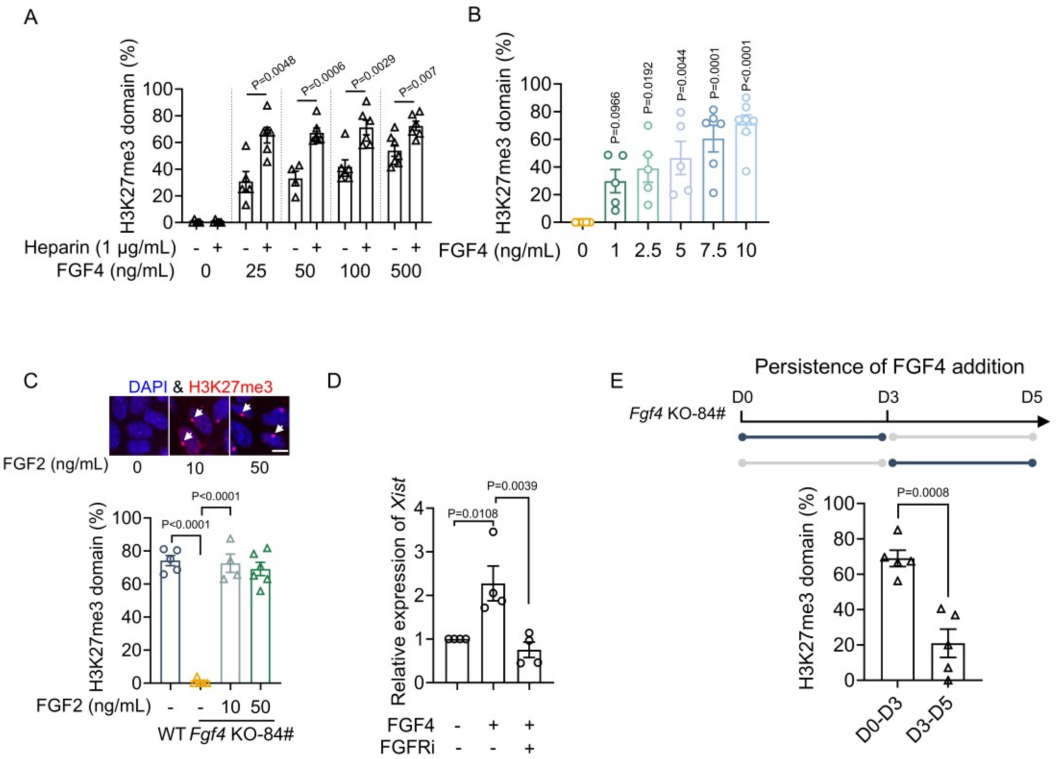

Appendix Figure S3. (A) Quantification of H3K27me3 domains in *Fgf4* KO-84# ES cells after addition of FGF4 and Heparin alone or in combinations on day 5 of differentiation; *n* = 5-7. (B) Quantification of H3K27me3 domains in *Fgf4* KO-84# ES cells after addition of 1, 2.5, 5, 7.5, 10 ng/mL FGF4 on day 5 of differentiation; *n* = 5-7. (C) Immunofluorescence staining and quantification of H3K27me3 domains in WT or *Fgf4* KO-84# ES cells after addition of 10 or 50 ng/mL FGF2 on day 5 of differentiation; *n* = 4-6; scale bars: 10 µm. The white in the nuclear H3K27me3 domain. % H3K27me3 = number of cells with nuclear H3K27me3 domains/ total number of analyzed cells. Cells with one H3K27me3 domain were considered as normal XCI and included in the statistical unit. (D) Relative expression levels of *Xist* in WT ES cells treated with 10 ng/mL FGF4 and FGF4 + nonspecific FGFR inhibitor (BGJ398, 75 nM) respectively on day 5 of differentiation; *n* = 4. (E) Immunofluorescence staining of H3K27me3 domains in *Fgf4* KO-84# ES cells after the addition of FGF4 at different time windows; *n* = 3. For the group D0-D3, cells cultured in differentiation medium N2B27 with 10 ng/ mL FGF4 for the first three days, and the medium was replaced by N2B27 without FGF4 to complete the differentiation process; For the group D3-D5, cells cultured in N2B27 without FGF4 for the first three days, and the medium was replaced by N2B27 with 10 ng/mL FGF4 to the end of differentiation; *n* = 5. (A-E) Data are shown as means ± SEM, *P* value was calculated by two-way ANOVA test with multiple comparisons (C and D), one-way ANOVA test with multiple comparisons (B) and unpaired two-tailed Student's t-test (A and E).

Appendix Figure S4

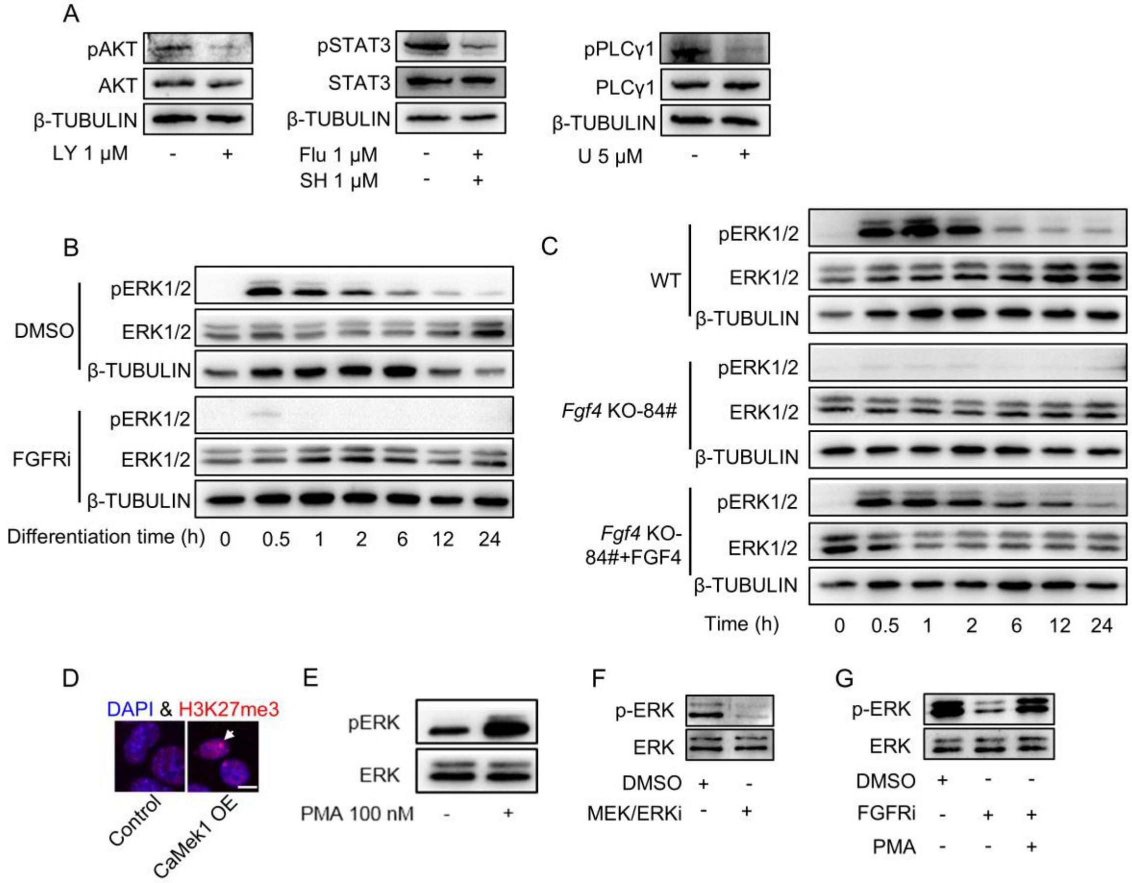

Appendix Figure S4. (A) Western blot analysis of phosphorylated and total AKT, STAT3 or PLCγ1 in WT ES cells treated with the inhibitor specific to PI3Ki, STATi and PLCγi on day 5 of differentiation. PLCγi: U73122 (U), 5 μM; STATi: Fludarabine (Flu), 1 μM plus SH-4-54 (SH), 1 μM; PI3Ki: LY294002 (LY) 1 μM. (B) Western blot analysis of phosphorylated and total ERK in WT ES cells treated with pan-FGFR inhibitor (FGFRi, BGJ398, 75 nM) at 0, 0.5, 1, 2, 6, 12 and 24 h of differentiation. (C) Western blot analysis of phosphorylated and total ERK in WT, *Fgf4* KO-84#, *Fgf4* KO-84#+FGF4 ES cells on 0, 0.5, 1, 2, 6, 12 and 24 h of differentiation. (D) Immunofluorescence staining of H3K27me3 domains in WT ES cells transfected with *Mek1* overexpression vector or empty vector on day 5 of differentiation; scale bars: 10 μm. The white in the nuclear H3K27me3 domain. % H3K27me3 = number of cells with nuclear H3K27me3 domains/ total number of analyzed cells. Cells with one H3K27me3 domain were considered as normal XCI and included in the statistical unit. (E) Western blot analysis of phosphorylated and total ERK in WT treated with 100 nM PMA on day 5 of differentiation. (F and G) Western blot analysis of phosphorylated and total ERK in *in vivo* E6.5 epiblast from females undergoing intraperitoneal injection with DMSO, FGFRi and FGFRi+PMA respectively; *n* = 7-17. FGFRi: BGJ398, 10mg/kg; PMA, 2.5mg/kg.

Appendix Figure S5

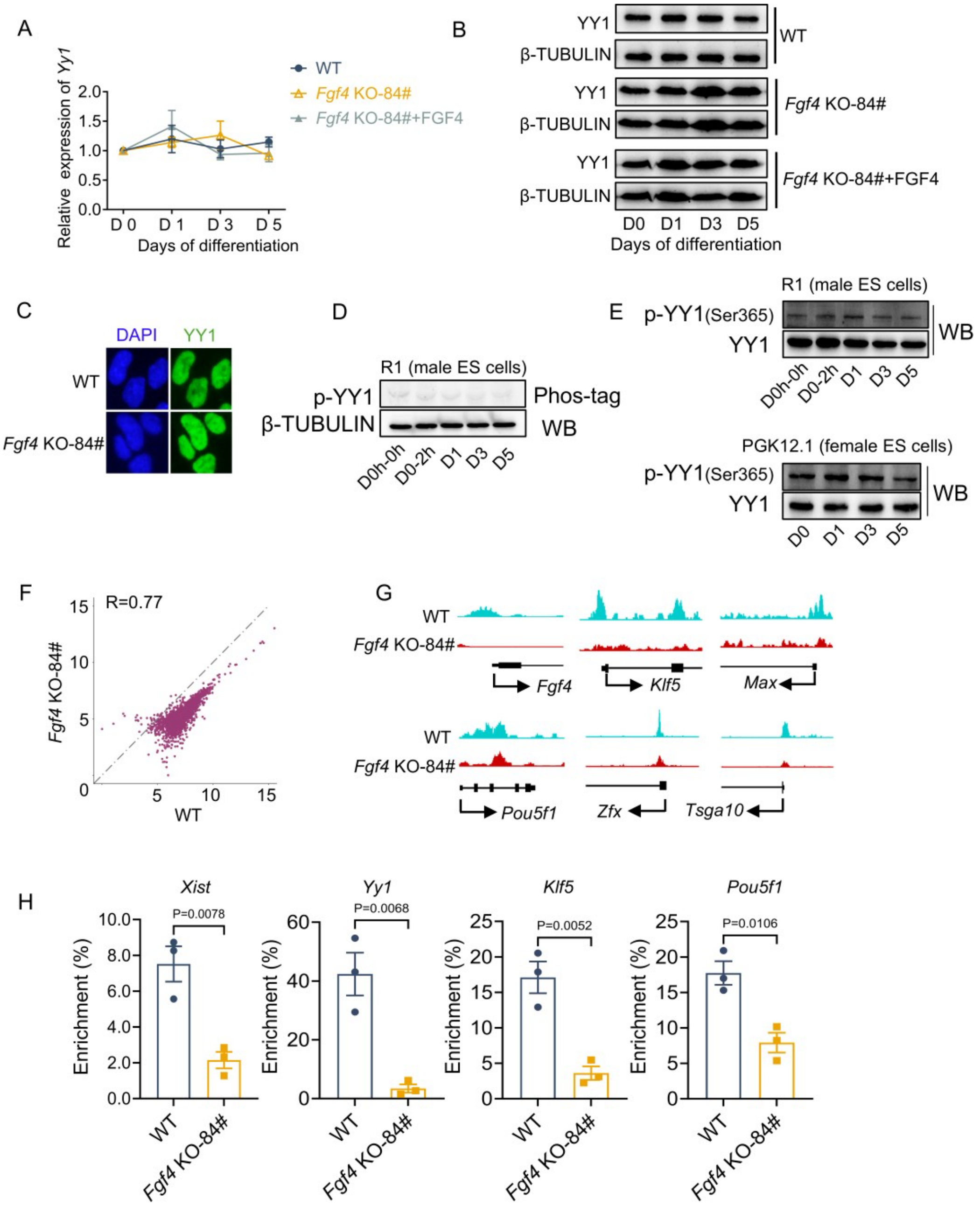

Appendix Figure S5. (A) Dynamic expression levels of *Yy1* in WT, *Fgf4* KO-84#, *Fgf4* KO-84#+FGF4 ES cells on day 0, 1, 3 and 5 of differentiation; *n* ≥ 6. (B) Western blot analysis of YY1 in WT, *Fgf4* KO-84#, *Fgf4* KO-84#+FGF4 ES cells on day 0, 1, 3 and 5 of differentiation. (C) Immunofluorescence staining of YY1 in WT and *Fgf4* KO-84# ES cells on day 5 of differentiation. (D) Phos-tag analysis of pYY1 and Western blot analysis of β-TUBULIN in male ES cells (R1) on day 0-0h, day 0-2h, day 1, day 3 and day 5 of differentiation. (E) Western blot analysis of p-YY1(Ser365) and total YY1 in male ES cells (R1) (upper panel) on day 0-0h, day 0-2h, day 1, day 3 and day 5 of differentiation and female ES cells (PGK12.1) (lower panel) on day 0, day 1, day 3 and day 5 of differentiation. (F) Scatter plots comparing the YY1 CUT&Tag signals (entire genome) between replicates (two biological replicates) for each stage. The Pearson correlation coefficients are also shown. (G)The UCSC browser view showing YY1 enrichment at representative promoters region in WT, *Fgf4* KO-84# ES cells. Gene models are shown below;

arrows indicate the direction of transcription. (H) CUT&RUN–qPCR enrichment of YY1 at the *Yy1*, *Xist*, *Klf5*, *Pou5f1* locus is shown as fold enrichment over IgG in WT and *Fgf4* KO-84# ES cells; *n*=3. Data are shown as means ± SEM, *P* value was calculated by unpaired two-tailed Student's t-test (H).

Appendix Figure S6

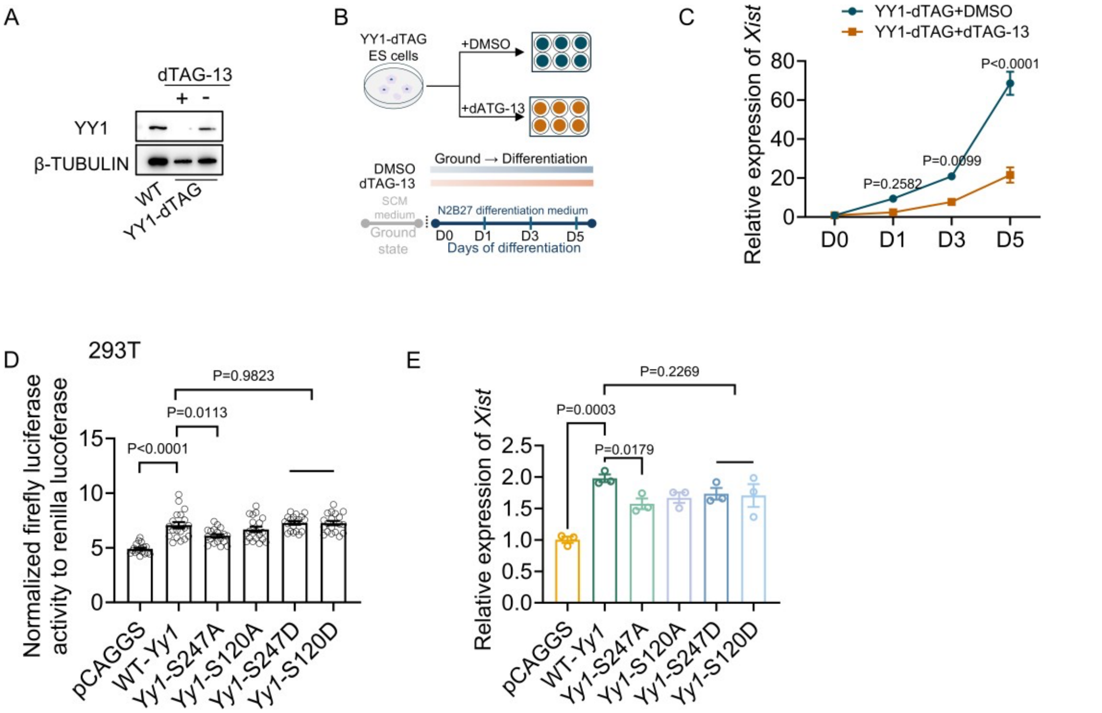

Appendix Figure S6. (A) Western blot analysis of YY1 protein levels in wild-type ES cells and YY1-dTAG ES cells at 24h after dTAG treatment or 24 hours after dTAG withdrawal. β-TUBULIN is used as a loading control. (B) Schematic showing the differentiation protocl for YY1-dTAG ES cells with DMSO or dTAG treatment. (C) Relative *Xist* expression levels between DMSO- and dTAG-treated ES cells at different time points, revealing the functional loss of wild-type endogenous YY1 on day 0, day 1, day 3 and day 5 of differentiation; *n*=3. (D) Relative luciferase activity of the reporters containing *Xist* promoter, which were co-transfected with an empty vector (pCAGGS), or overexpression vector of WT or mutant *Yy1* in 293T cells; *n* ≥ 6. Each dot indicates the result of each reaction and assays were replicated independently at least three times. (E) Relative expression levels of *Xist* in wild type ES cells transfected with an empty vector (pCAGGS), or overexpression vector of WT or mutant Yy1 on the YY1-depleted background; *n*=3. (C-E) Data are shown as means ± SEM, *P* value was calculated by two-way ANOVA test with multiple comparisons (C) and one-way ANOVA test with multiple comparisons (D and E).

Appendix Figure S7

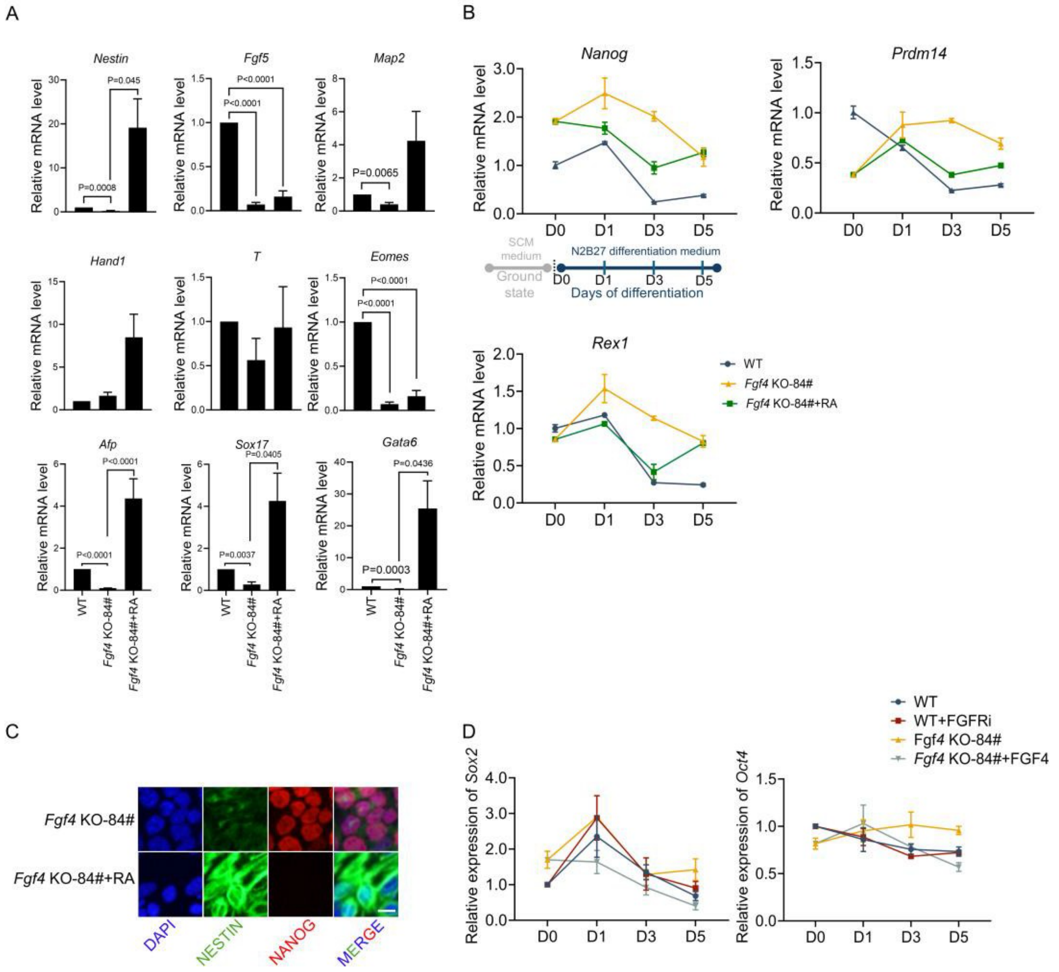

Appendix Figure S7. (A) Relative mRNA expression levels of marker genes specific to three germ layer lineages in WT, *Fgf4* KO-84#, *Fgf4* KO-84#+RA ES cells on day 5 of differentiation. (B) Dynamic expression levels of *Nanog*, *Prdm14*, *Rex1*, *Sox2*, *Oct4* in WT, *Fgf4* KO-84#, *Fgf4* KO-84#+RA ES cells on day 0, 1, 3 and 5 of differentiation; *n* = 3. The schematic diagram below the X-axis indicates the timeline of differentiation. The day when ES cells were transferred to the differentiation medium was designated as day 0, normalized to the WT ES cells control. (C) Immunofluorescence staining of NANOG and NESTIN in *Fgf4* KO-84#, *Fgf4* KO-84#+RA ES cells on day 5 of differentiation; scale bars: 10 μm. (D) Dynamic expression level of *Sox2* (C), *Oct4* (D) in WT, WT+FGFRi, *Fgf4* KO-84#, *Fgf4* KO-84#+FGF4 on day 0, 1, 3 and 5 of differentiation, shown relative to the WT control; *n*=3. (B and D) Data are shown as means ± SEM, *P* value was calculated by one-way ANOVA test with multiple comparisons (A).

References:

Barros DAES, Jonkers I, Syx L, Dunkel I, Chaumeil J, Picard C, Foret B, Chen CJ, Lis JT, Heard E, et al (2019) Kinetics of Xist-induced gene silencing can be predicted from combinations of epigenetic and genomic features. *GENOME RES* **29**(7)**:** 1087-1099

Schulz EG, Meisig J, Nakamura T, Okamoto I, Sieber A, Picard C, Borensztein M, Saitou M, Bluthgen N, Heard E (2014) The two active X chromosomes in female ESCs block exit from the pluripotent state by modulating the ESC signaling network. *CELL STEM CELL* **14**(2)**:** 203-216

Appendix Table S1. DNA oligonucleotides used in this study.

Primer sequences of genes for RT-qPCR

Gene	Primer sequence
Fgf4	F: TGGGCCTCAAAAGGCTTCG
	R: CGTCGGTAAAGAAAGGCACAC
Fgfr1	F: TGGACCGCATTGTGGCCTTGAC
	R: TCAGCGCCGTTTGAGTCCACTG
Fgfr2	F: TGCACGCAGGATGGACCTCTCT
	R: TGCTCCTCGGGGACACGGTTAA
Xist	F: GGCGGTGCAAACTAAAACTC
	R: CAGTAGGCTTAGAGAACCGC
Nanog	F: AGGATGAAGTGCAAGCGGTG
	R: TGCTGAGCCCTTCTGAATCAG
Oct4	F: CCCCAATGCCGTGAAGTTG
	R: TCAGCAGCTTGGCAAACTGTT
Sox2	F: CACAGATGCAACCGATGCA
	R: GGTGCCCTGCTGCGAGTA
Nestin	F: GGCATCCCTGAATTACCCAA
	R: AGCTCATGGGCATCTGTCAA
Map2	F: TTCTTTTGCTTGCTCGGGATT
	R: ATACAGGGCTTGGTTTATTTCAGAGA
Hand1	F: CGCCTGGCTACCAGTTACAT
	R: GGCCTGGTCTCACTGGTTTA
T	F: AAGGAACCACCGGTCATC
	R: GTGTGCGTCAGTGGTGTGTAATG
Eomes	F: CCCTATGGCTCAAATTCCAC
	R: CCCTATGGCTCAAATTCCAC
Afp	F: ATCACACCCGCTTCCCTCATCC
	R: TTCATTGCAGCCAACACATCGC
Sox17	F: ACTTGCTCCCCACAATCACT
	R: ACCCCGCTGTTTGTGTTTAG
Gata6	F: CTTCTCCTTCTACACAAGCGACCA
	R: ATACTTGAGGTCACTGTTCTCGGG
Yy1	F: GTGGTTGAAGAGCAGATCATTGG
	R: TTGCTTAGGGTCTGAGAGGTC
Gapdh	F: TGCCCCCATGTTTGTGATG
	R: TGTGGTCATGAGCCCTTCC

Primer sequences of genes for genotyping

Gene	Primer sequence
Fgf4	F: GTCCCTATTTGCTCTCGCTACT
	R: GTCTCCTATCCGGTAGGTCCA
Yy1	F: CTGTGCAGTGATTGGGTCCT
	R: TTGCCGCTCTGCACTTAAGT

Primer sequences of genes for ChIP-qPCR and CUT&RUN-qPCR

Gene	Primer sequence
YY1-*Xist-1*	F: ACGGCTATTCTCGAGCCAGTT
	R: ATTGGTTGCTTTTATCCAGT
YY1-*Xist-2*	F: GTCTATAAAATGGCGGCTCG
	R: CGCCATCTTTTCCTGTACG
YY1-*Klf5*	F: CTCCATTGTCCTAGGGATGCC
	R: ACTTTATTCACGGGGCTCCAG
YY1-*Pou5f1*	F: GGTGAGAGGACCTTGAAGGTTGA
	R: CTAGGGACGGTTTCACCTCTCCC
YY1-*Yy1*	F: TCGCCTGATCGACCTAGAAC
	R: CATAGGGCCCACGACTTAAA

Primer sequences of genes for sgRNA

Gene		Primer sequence	
Fgf4	1	F: CACCGCGAGCTGCGGGCCGTGAGTT	R: AAACAACTCACGGCCCGCAGCTCGC
		F: CACCGTCACGCACGGCCCGCGGGCC	R: AAACGGCCCGCGGGCCGTGCGTGAC
	2	F: CACCGACGGCAGAATGCTCCCGATC	R: AAACGATCGGGAGCATTCTGCCGTC
		F: CACCGTGGTATGAACCCTGTCGGAT	R: AAACATCCGACAGGGTTCATACCAC
Nanog	1	F: AAACGTGTCCCGGATCTCTATTTC	R: CACCGAAATAGAGATCCGGGACAC
		F: CACCGCTAGAAGGCCAACGTATCAC	R: AAACGTGATACGTTGGCCTTCTAGC
	2	F: AAACGGAAGACCCACACTCATGTC	R: CACCGACATGAGTGTGGGTCTTCC
		F: CACCGTCATGTCAGTGTGATGGCGA	R: AAACTCGCCATCACACTGACATGAC
Prdm14	1	F: CACCGCCGGCATTCCTTAGTCAAGG	R: AAACCCTTGACTAAGGAATGCCGGC
		F: CACCGCCGCTCCCAAGCCCTCAACC	R: AAACGGTTGAGGGCTTGGGAGCGGC
	2	F: CACCGATGTGCATGAGTGTTCGCCG	R: AAACCGGCGAACACTCATGCACATC
		F: CACCGCTACAGACACCGTATAACG	R: AAACCGTTATACGGTGTCTGTAGC
Rex1	1	F: CACCGCAGGATCAAACTGAGCTGAG	R: AAACCTCAGCTCAGTTTGATCCTGC
		F: CACCGATCCTGGGATACGTGTGGC	R: AAACGCCACACGTATCCCAGGATC
	2	F: CACCGCGAGCTCATTACTTGCAGG	R: AAACCCTGCAAGTAATGAGCTCGC
		F: CACCGAGCTCGCCCCAACCCTCAG	R: AAACCTGAGGGTTGGGGCGAGCTC
Yy1		F: CACCGTCTTCTCTCTTCTTTTCAC	R: AAACGTGAAAAGAAGAGAGAAGAC

Primer sequences of genes for plasmids construction

Gene	Primer sequence
oe-*Yy1*	F: CCGGAATTCGCCACCATGGCCTCGGGCGACACCCTC
	R: CCGCTCGAGCTGGTTGTTTTTGGCTTTAGC
Yy1-S247A	F: AGATCATTGGAGAGAACCGACCTCCTGATTATTCTG
	R: CGGTTCTCTCCAATGATCTGCTCTTCAACCACTGT
Yy1-S120A	F: GTGGTCGGCGGGGACGACCGGGACGGGCTGCGCGCC
	R: CGGTCGTCCCCGCCGACCACCTCCTCGCGCGTCTGC
Yy1-S247D	F: AGATCATTGGAGAGAACCTACCTCCTGATTATTCTG
	R: AGGTTCTCTCCAATGATCTGCTCTTCAACCACTGT
Yy1-S120D	F: GTGGTCGGCGGGGACGACCTGGACGGGCTGCGCGCC
	R: AGGTCGTCCCCGCCGACCACCTCCTCGCGCGTCTGC
Xist-Promoter	F: GGTCGGAGCTCGTTTTCTTGGAGTCATTTACTGCAGTCTT
	R: GGTGCAAGCTTGGCTAAATCAGAGGCCAAGGTGTAA
